# Ginkgolide B Exerts Cardioprotective Properties against Doxorubicin-Induced Cardiotoxicity by Regulating Reactive Oxygen Species, Akt and Calcium Signaling Pathways *In Vitro* and *In Vivo*

**DOI:** 10.1371/journal.pone.0168219

**Published:** 2016-12-14

**Authors:** Junqing Gao, Tao Chen, Deqiang Zhao, Jianpu Zheng, Zongjun Liu

**Affiliations:** Department of Cardiology, Putuo Hospital, Shanghai University of Traditional Chinese Medicine, Shanghai, People’s Republic of China; State University of New York, UNITED STATES

## Abstract

The aim of this study was to evaluate the effect of Ginkgolide B (GB) on doxorubicin (DOX) induced cardiotoxicity *in vitro* and *in vivo*. Rat cardiomyocyte cell line H9c2 was pretreated with GB and subsequently subjected to doxorubicin treatment. Cell viability and cell apoptosis were assessed by MTT assay and Hoechst staining, respectively. Reactive oxygen species (ROS), Akt phosphorylation and intracellular calcium were equally determined in order to explore the underlying molecular mechanism. To verify the *in vivo* therapeutic effect of GB, we established a mouse model of cardiotoxicity and determined left ventricle ejection fraction (LVEF) and left ventricular mass (LVM). The *in vitro* experimental results indicated that pretreatment with GB significantly decreases the viability and apoptosis of H9c2 cells by decreasing ROS and intracellular calcium levels and activating Akt phosphorylation. In the *in vivo* study, we recorded an improved LVEF and a decreased LVM in the group of cardiotoxic rats treated with GB. Altogether, our findings anticipate that GB exerts a cardioprotective effect through possible regulation of the ROS, Akt and calcium pathways. The findings suggest that combination of GB with DOX in chemotherapy could help avoid the cardiotoxic side effects of GB.

## Introduction

Doxorubicin (DOX), a potent anthracycline antibiotic, is widely recognized as an effective chemotherapeutic agent used in the therapy of different types of cancer in clinical settings [1–9]. However, regrettably, several studies have reported that DOX induces dose-dependent acute or chronic cardiotoxicity [10–15] through a variety of mechanisms involving increased cardiac oxidative stress, changes in adenylate cyclase activity, lipid peroxidation, and the activation of inflammation and apoptosis-related signaling pathways [16, 17], leading to late-onset cardiomyopathy in a dose cumulative manner [18].These cardiotoxic effects constitute a key drawback of DOX-based chemotherapy [19].

Due to the relevance and the efficacy of DOX in cancer chemotherapy, strategies for preventing or attenuating the side effects of DOX administration, including the alternative drugs with antagonistic properties against DOX induced cardiotoxicity, nanoparticle co-delivery system, and the iron-chelating agents [20–29] have been attempted. Nevertheless, definitively efficient drugs to against DOX-cardiotoxicity have not been developed so far and the discovery of novel agents for thwarting its side effects is still encouraged.

In recent years, numerous research works have indicated that extracts of *Ginkgo biloba* leaves may be beneficial for preventing from the drug-induced toxicity on non-tumour tissues such as the liver, lung, kidney, and heart due to its various pharmacological properties, including anti-inflammatory effect, anti-tumor effect, anti-apoptotic effect, and antioxidant activity [30–35].

Ginkgolide B (GB) is the major terpenoid component extracted from *G*. *Biloba* leaves. Previous studies have suggested that GB could exert an antagonistic activity against the platelet activating factor (PAF) to subsequently inhibit PAF-induced cascade effect in inflammatory reactions [36–38]. Most recently, researchers have discovered that GB exerts modulatory or protective functions by reducing oxidative stress and Aβ-induced dysfunction of mitochondrial oxidative phosphorylation of the neuronal cells and maintaining cellular energy demands [39]. However, surveys on the effect of GB on DOX-induced cardiotoxicity and the potential molecular mechanisms are limited and need an in-depth elucidation.

Thus, the present study was designed to examine the potential protective effect of GB against DOX-induced cardiotoxicity and to give insights into its possible underlying molecular mechanisms. Specifically, we studied the effect of GB pretreatment on the viability of cardiomyocytes challenged with DOX *in vitro* and its cardio-protective effects *in vivo* and found that GB could protect against DOX induced cell death in H9c2 cardiomyocytes and improved cardiac function *in vivo*.

## Methods

### Cell culture

Rat H9c2 cells were purchased from American Type Culture Collection (ATCC, Manassas, VA) and cultured in high-glucose DMEM media containing 10% fetal bovine serum (FBS), 100 U/ml penicillin and 100 μg/ml streptomycin. Cells were cultured in a 5% CO_2_ incubator at 37°C and were split when 80% confluence was reached.

### Cell viability assay

Cardiomyocytes (4×10^3^ cells) were suspended in 100 μL culture media and seeded in triplicate in 96-well plate overnight. Thereafter, cell cultures were incubated with a series of different concentrations (0–10 μM) of DOX (Sigma-Aldrich Co.Ltd; St. Louis, MO, USA) for 72 h in the presence or absence of indicated concentrations (0–100 mM; dissolved in DMSO) of GB (sc-201037, Santa Cruz Biotechnology, Santa Cruz, CA) for 24 h. The purity of GB was more than 90%. In the setting of verification of the protective role of GB, cardiomyocytes were either pretreated with 1, 5 and 50 μM GB for 30 min before being challenged with 5 μM DOX or treated with 5 μM DOX 3 h before incubating with 1, 5 and 50 μM GB. In the investigation of molecular mechanisms, cardiomyocytes were pretreated with 50 μM GB or small molecular inhibitors targeting given metabolic pathways (LY294002 (10 μM) or KN62 (10 μM)) for 30 min before being challenged with 5 μM DOX. After 48 h incubation, 3-(4,5-dimethylthiazolyl-2)-2,5-diphenyltetrazolium bromide (MTT: 0.5 mg/ml; Sigma, MO, USA)[40–42] was added and further incubated for 4 h, followed by addition of formazan dye dissolved with DMSO in a microplate reader (Biotek Instruments, Winooski, VT) and reading at 570 nm. Cell viability expressed as relative viability compared with control in each experiment. All the experiments were performed in triplicate.

### Hoechst nuclear staining

Double nuclear staining with fluorescent dyes Hoechst 33258 (Beyotime, Nantong, China) was used to determined cell apoptosis according to a previous protocol [43]. Briefly, after the cells were incubated with different treatment for 24 h, Hoechst 33258 was added into the culture medium at a concentration of 8 μM, and incubated at 4°C for 30 min. Images were captured by a confocal microscope and cells in 5 randomly picked fields (400× magnification) were counted, and the percentage of apoptotic cells were calculated.

### Annexin V/propidium iodide staining

The H9c2 cardiomyocytes were processed with Annexin V/propidium iodide (PI) staining according to the manufacturer’s instructions (Beyotime, Nantong, China). The samples were analyzed by a FACSCalibur flow cytometry (Becton Dickinson, Franklin Lakes, NJ).

### Immunofluoresence analysis

After different treatments, cardiomyocytes were fixed with 4% paraformaldehyde. Cells were stained with rat anti phospho-Akt antibody (Cell Signalling Technology) and then with secondary antibody Alexa Fluor® 555 (Invitrogen). Simultaneously, cellular nuclei were stained with 4'.,6-diamidino-2-phenylindole 4′,6-Diamidino-2-phenylindole (DAPI, Molecular Probes, Carlsland, CA). After secondary labelling, cells were washed with PBS and fixed with ProLong® Gold antifade reagent. Fluorescence microscopy was used to determine the fluorescence signals (Olympus, Japan).

### Western-blot analysis

Total proteins were extracted from cardiomyocytes as described elsewhere [44], subsequently separated by electrophoresis on SDS-PAGE gel and then transferred onto PVDF membrane. After blocking, the blots were incubated with the antibodies against Caspase-3 (Abcam, Cambridge, MA), phosporylated or total Akt (Millipore, Beverly, MA), p38 (Santa Cruz Technology, Santa Cruz, CA), JNK (Cell signaling Technology, Beverly, MA), Erk (Cell signaling Technology) or CamKII (Cell signaling Technology). β-actin (Abcam) was used as loading control. Following, samples where incubated with appropriate HPR conjugated secondary antibodies. The protein bands were revealed using the SuperSignal Ultra Chemiluminescent Substrate (Pierce, Rockford, IL USA) on X-ray films (Koda, Lexington, MA, USA) followed by data quantification processing using Image J software (NIH, Bethesda, MD, USA).

### Assessment of intracellular Ca^2+^

After the cultured H9c2 cardiomyocytes adhered to the chamber cover-slips, the standard Tyrode's solution (126 mM NaCl, 5.4 mM KCl, 10 mM HEPES, 0.33 mM NaH_2_PO_4_·2H_2_O, 1.0 mM MgCl_2_·6H_2_O, 1.8 mM CaCl_2_ and 10 mM Glucose; pH = 7.40) was used to rinse cells. Thereafter, cells were incubated with a working solution containing Fura-2/AM (20 mM, Beyotimes, Nantong, China) and Pluronic F-127 (0.03%) at 37°C for 45 min. Once loaded, the above standard Tyrode's solution was used again to wash cells and eliminate the extracellular fluo-3/AM. The concentration of Ca^2+^ was measured using Ca^2+^ indicator fluo-3/AM as probe. The images were captured using a 20× objective (488 nm excitation, 530 nm emission). Scanning time was 30 min. Reagents were added between the 3rd and 4^th^ scans (10 s interval) and the images were saved.

### Detection of reactive oxygen species (ROS)

Cardiomyotes were pretreated with 5 μM GB and incubated with 5 μM DOX for 24 h. After that, ROS was induced by treatment with retigeric acid B (RAB). Subsequently, flow cytometry was employed for detecting ROS using the fluoprobe hydroethidine 2’,7’-Dichlorodihydrofluorescein diacetate (H2DCFDA, Beyotimes, Nantong, China) according to the manufacturer’s instructions. Briefly, around 1×10^6^ PBS-washed cells of each treatment group were loaded with 0.5 ml PBS containing 10 μM of H2DCFDA and incubated in the darkness for 40 min. The parameter settings for flow cytometry were: emission wavelength = 535 nm, excitation wavelength = 488 nm. The ROS level was proportionally deduced from the fluorescence intensity of the oxidized product of H2DCFDA (2', 7'-dichlorofluorescein).

### Animal studies

#### Ethics statement

The animal study protocol was approved by the institutional animal care and use committee of the Putuo Hospital affiliated to Shanghai University of Traditional Chinese Medicine and was performed in accordance with the generally accepted international guidelines for animal experimentation. The animals were maintained in appropriate cages at 22±1°C on a 12 h light/dark cycle with free access to food and water, and were allowed to acclimatize for a minimum of 1 week before the experiment. Efforts were made to minimize pain by isoflurane anesthesia.

#### *In vivo* mouse model of DOX-induced cardiotoxicity

C57BL/10 mice (8- to 10-week-old) were randomly divided into 4 groups (*n* = 6 per group). Two groups received DOX (Santa Cruz Technology; 20 mg/kg; i.p.) at a dose that had been shown to be cardiotoxic [45]. Four days before DOX application, in one DOX group, a treatment with GB (100 mg/kg/day, i.p.; Sigma-Aldrich) was started. The other DOX group received saline. The other two groups without DOX application received no further treatment or the same GB as above. Five days after DOX injection, the Vevo770TM imaging system (VisualSonics Inc., Toronto, Canada) was used to measure mice electrocardiograms (ECGs). The left ventricle ejection fraction (LVEF) was calculated according to a previous protocol [46, 47]. Briefly, mice were put under isoflurane anesthesia and a rectal probe and an infrared heating lamp were respectively used to monitor and control the body temperature. Electrode pads on the heated platform was used to monitor the ECG signal. Chest hair was removed using razor and a chemical depilator to minimize ultrasound attenuation. By using the warm ultrasound gel, we placed the ultrasound probe (RMV-707B) on the chest of the mice. Two-dimensional images were taken in the parasternal short- and long- axis views to direct the M-mode records obtained at the mid-ventricular level, using 3 to 5 measurements for each view and the mean was calculated. The LV systolic function was calculated based on the M-mode measurements following the recommendations of the American Society of Echocardiography Committee. After the echocardiography, all the animals were euthanatized with an overdose of sodium pentobarbital (i.p.).

### Statistical analysis

All the statistical analyses were performed using GraphPad Prism software version 6. The data was presented as Mean ± S.E.M. One way-ANOVA was used to examine the difference in three or more groups followed by Tukey's post-hoc test. P<0.05 was considered for statistical significance.

## Results

### GB protects from DOX-induced cardiac cell death

**Fig 1:**
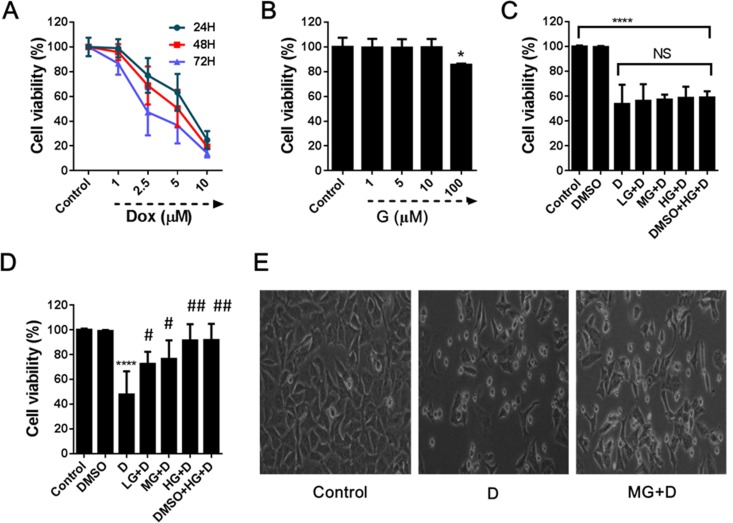
Protective effect of GB against DOX-induced cardiac cell death. (**A**) Time and dose-dependent DOX-induced cardiac cell death was determined by MTT assay. (**B**) The safety dosage of GB on the cardiomyocytes viability was assessed by MTT assay. (**C**) Cardiomyocytes were treated with low (LG, 1μM) medium (MG, 5 μM) and high (HG, 50 μM) of GB after DOX treatment for 3 h and no improvement was found on the cardiomyocytes viability in DOX induced cardiotoxicity. (**D**) Pretreatment with low (LG), medium (MG) and high (HG) doses of GB for 30 min prior to DOX-induced cardiotoxicity increased cardiomyocytes viability. (**E**) Representative images of the effects of DOX and pretreatment with GB for 30 min on cardiomyocytes proliferation. ****p<0.0001 when compared to control group, #p<0.01, ##p<0.001 when compared to DOX.

Prior to the investigation of the effect of GB on DOX-induced cardiotoxicity, we measured the effect of different concentrations of these agents on the viability of rat H9c2 cells in order to determine the optimal concentration of DOX and safety dosage for GB treatment. The results showed that 5 μM DOX could achieve an appropriate degree of cell death after 48 h (Fig 1A) and the viability of cardiomyocytes was not affected up to 50 μM GB (Fig 1B). Thereafter, we employed two models to test the protective effect of GB against DOX cardiotoxicity and found that in the model of cardiomyocytes treatment with DOX for 3 h before GB application, GB treatment did not reverse the toxic effect of DOX on cardiomyocytes (Fig 1C). On the contrary, in the model that cardiomyocytes were pretreated with GB for 30 min before DOX incubation, significantly increased cell viability was observed (Fig 1D). Microscopic examination revealed results similar to that of the MTT assay (Fig 1E).

### GB protects from DOX-induced cardiac cell apoptosis

**Fig 2:**
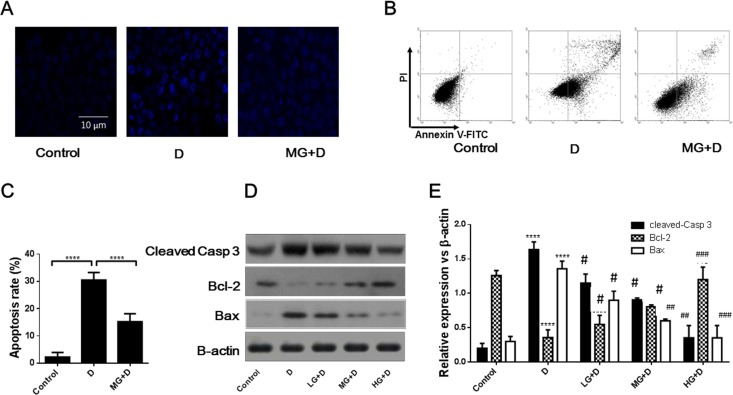
Protective effect of GB against DOX induced cardiomyocytes apoptosis. (**A**) Representative images of Hoechst nuclear staining in each group. The extent of nuclear damage was decreased in the group of GB pretreatment for 30 min followed by DOX-induced cardiotoxicity comparatively to the DOX group. (**B**) Flow cytometry analysis of the cardiomyocytes apoptosis. Protective effects of GB pretreatment on the apoptosis of cardiomyocytes were demonstrated by Annexin V/PI staining. (**C**) Quantitative representation of the flow cytometry analysis. Cardiomyocytes apoptosis was inhibited following GB pretreatment for 30 min before DOX-induced cardiotoxicity. (**D**) and (**E**) Western-blot analysis of apoptosis related proteins (cleaved caspase 3, Bcl-2 and Bax) in cardiomyocytes after GB pretreatment for 30 min prior to DOX-induced cardiotoxicity. ****p<0.0001 when compared to control group, #p<0.05 ##p<0.01, ###p<0.001 when compared to the DOX group.

To determine the extent of cell apoptosis, we first performed Hoechst nuclear staining and found that 5 μM GB pretreatment significantly decreased cell death (Fig 2A). Further, Annexin V/PI staining indicated that GB pretreatment significantly reduced DOX-induced cardiac cell apoptosis (Fig 2B and 2C). The measurement of the effect of pretreatment with different GB dosages on the expression of apoptotic markers by western blotting showed that GB induces the expression of Bcl-2 and repressed the expression of Bax in a dose-dependent manner (Fig 2C and 2D). The cleaved capase-3 was also evaluated and the results showed that GB pretreatment noticeably decreased the level of cleaved-caspase-3 (Fig 2D), which is indicative of reduced cell apoptosis.

### ROS was involved in the protective effect of GB

**Fig 3:**
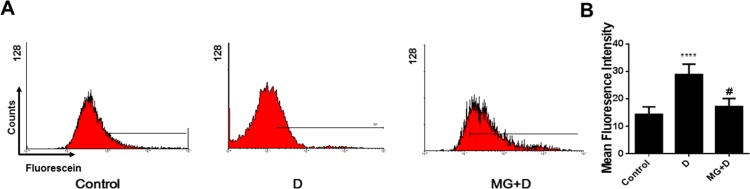
GB pretreatment decreased the level of reactive oxygen species (ROS) in cardiomyocytes treated with DOX. (**A**) Flow cytometry analysis of ROS level in cardiomyocytes treated with 5 μM GB (MG) before subjection to DOX induced cardiotoxicity. (**B**) Quantification of the mean fluorescence intensity (MFI) obtained from Flow cytometry. ****p<0.0001 when compared to control group, #p<0.0001 when compared to DOX.

The reactive oxygen species (ROS) is reported to be in involved in the DOX-induced cardiotoxicity. To examine whether GB exerts its protective effect through ROS, we performed flow cytometry analysis for determination of ROS levels. The results showed that DOX treatment increased ROS levels while GB pretreatment significantly inhibited ROS expression in DOX-treated mice (Fig 3). These results indicate that GB exerts its protective effects via downregulation of ROS.

### Intracellular calcium level and CaMKII phosphorylation was involved in the protective effect of GB pretreatment

**Fig 4:**
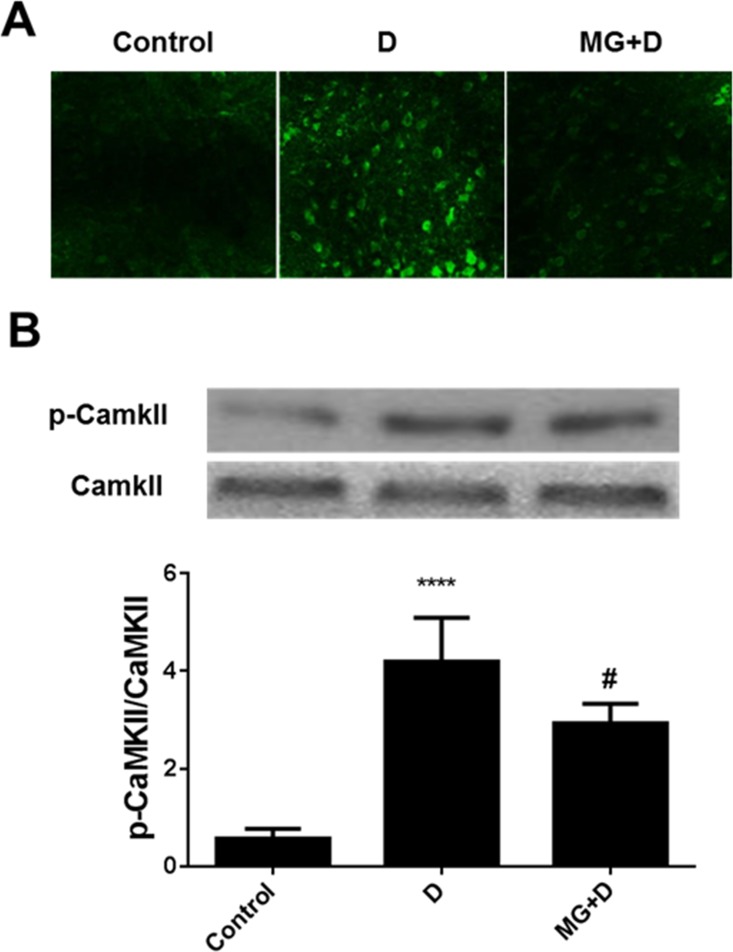
Intracellular calcium level and CaMKII phosphorylation was involved in the protective effect of GB in DOX induced cardiotoxicity. (**A**) Intracellular calcium level was determined by Fura-2/AM probe. Increased intracellular calcium level was found after DOX treatment while GB pretreatment could significantly decrease the calcium level (**B**). Western blot analysis of CaMKII phosphorylation. Increased CaMKII phosphorylation was found after DOX treatment while GB pretreatment could significantly decrease the CaMKII phosphorylation. ****p<0.0001 when compared to control group, #p<0.0001 when compared to DOX.

Fura-2/AM probe was employed to examine the intracellular calcium level. We observed that GB pretreatment could reverse the DOX-induced upregulation of calcium levels induced by treatment (Fig 4A). Moreover, we observed the effect of GB pretreatment could significantly decrease CaMKII phosphorylation (Fig 4B).

### Akt phosphorylation was involved in the protective effect of GB

**Fig 5:**
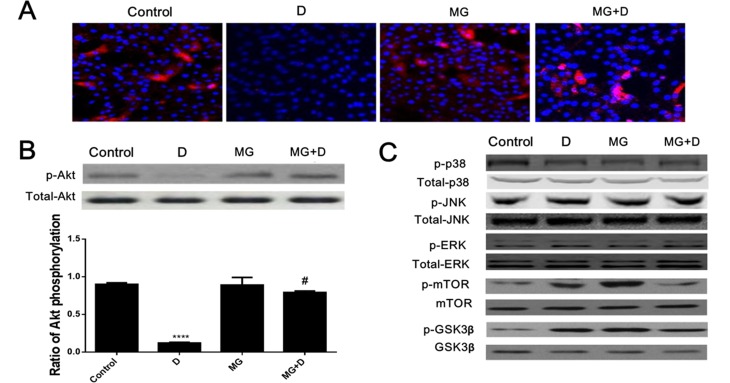
Akt phosphorylation was involved in the protective effect of GB in DOX induced cardiotoxicity. (**A**) Immunofluorescence analysis of Akt phosphorylation. Decreased Akt phosphorylation was found after DOX treatment but pretreatment with GB restored this effect. (**B**) Western-blot analysis of Akt phosphorylation. Significantly decreased Akt phosphorylation was found after DOX treatment but GB treatment significantly activated the Akt phosphorylation. (**C**) Phosphorylation of p38, JNK, Erk, mTOR and GSK3β was not involved in the protective effect of GB in DOX induced cardiotoxicity. No significant difference was found on p38, p-JNK, p-Erk, p-mTOR and p-GSK3β as detected by western-blot analysis. ****p<0.0001when compared to control group, #p<0.0001 when compared to DOX.

We further performed the immunofluoresence (Fig 5A) and western-blot analysis (Fig 5B) to assess the role of Akt phosphorylation in the protective effect of GB pretreatment. GB pretreatment could reverse the downregulation of Akt phosphorylation induced by DOX. Moreover, GB pretreatment did not affect the phosphorylation of p38, JNK, Erk, mTOR and GSK-3β (Fig 5C).

#### Inhibition of CaMKII and Akt pathways protects against DOX-induced cardiotoxicity

**Fig 6:**
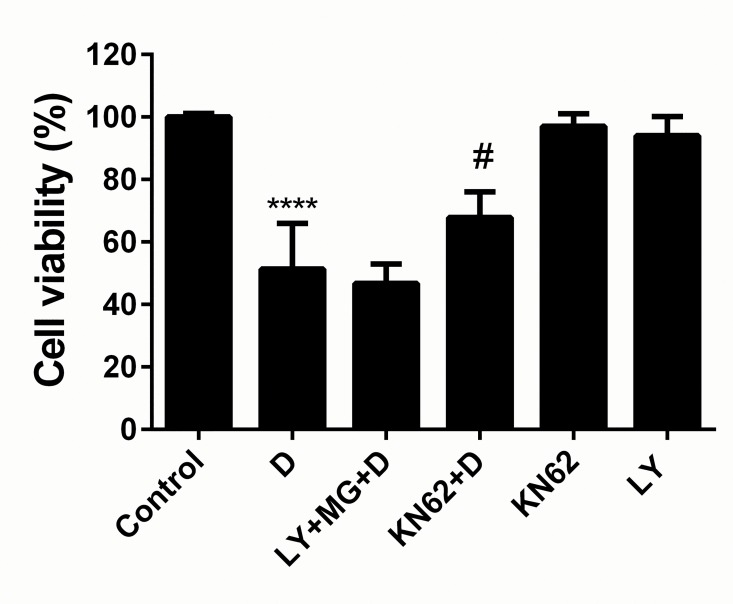
GB exert its protective effect by activating the PI3K/Akt pathway and inhibiting the CaMKII pathway. Cardiomyocytes cell viability analysis was determined by MTT assay. PI3K inhibitor LY294002 (10 μM) pretreatment inhibited the protective effect of GB pretreatment in DOX induced cardiotoxicity while CaMKII inhibitor KN62 (10 μM) pretreatment significantly increased cell viability. ****p<0.0001 when compared to control group, #p<0.01 when compared to DOX group.

Due to the effects of GB on CaMKII and Akt phosphorylation, we aimed to verify if inhibition of these pathways could affect cell viability. Cardiomyocytes were treated with PI3K inhibitor LY294002 and CaMKII inhibitor KN62 and the cell viability measured. The results showed that PI3K inhibitor LY294002 pretreatment could reverse the protective effect of GB in DOX-induced cardiotoxicity while CaMKII inhibitor KN62 pretreatment significantly increased cell viability (Fig 6). These results suggest that GB exert its protective effect by activating the PI3K/Akt pathway and inhibiting the CaMKII pathway.

### Protective effect of GB on cardiac function in DOX induced cardiotoxic model *in vivo*

**Fig 7:**
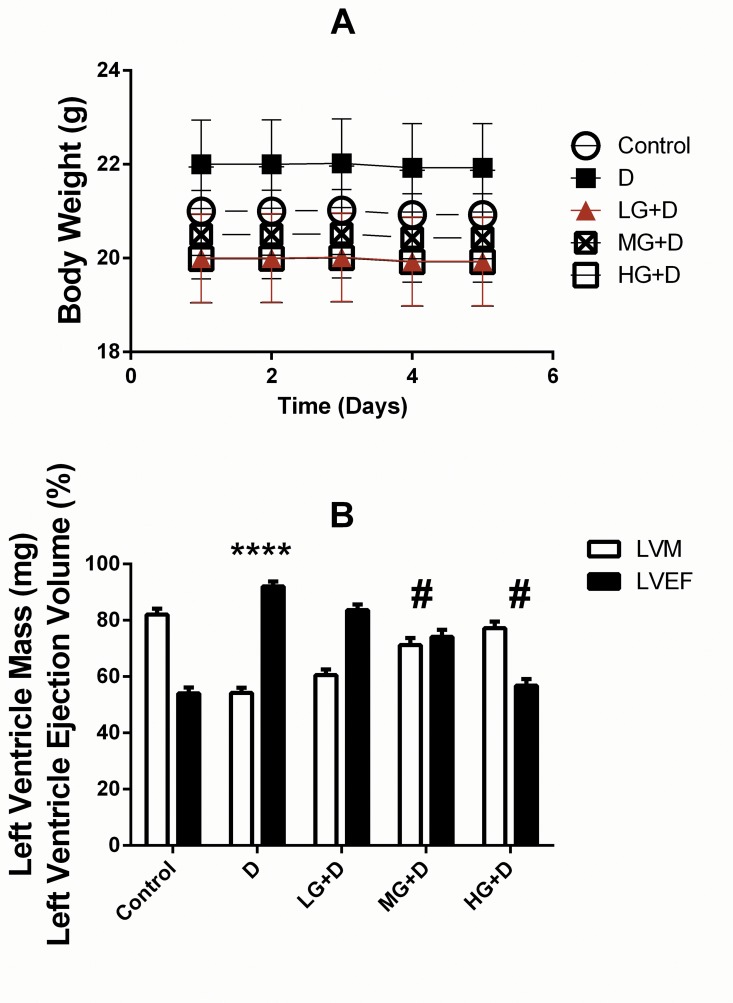
Protective effect of GB on cardiac function in DOX induced cardiotoxic model *in vivo*. (**A**) No significant difference was recorded regarding mouse weight in mice treated with DOX, GB or both. (**B**) Significant improvement of the left ventricle ejection fraction (LVEF) and decreased left ventricle mass (LVM) were found in mice treated with GB in DOX-induced cardiotoxic model. ****p<0.0001 when compared to control group, #p<0.05 when compared to DOX.

In the *in vivo* model, no significant difference was found on the mouse weight in mice treated with DOX, GB or both (Fig 7A). On the contrary, significant improvement of the left ventricle ejection fraction LVEF and decreased left ventricle mass (LVM) in mice treated with GB in DOX induced cardiotoxic model (Fig 7B).

## Discussion

In the present study, we investigated the protective effects of GB on DOX-induced cardiotoxicity and found that GB pretreatment significantly improved cell viability by inhibiting cell apoptosis. We equally found that GB pretreatment decreased ROS and intracellular calcium levels and increased Akt phosphorylation in the cardiotoxic cell model. In the *in vivo* carditoxic model, we found improved LVEF and decreased LV mass in the mice treated with GB. To the best of our knowledge, our study is the first to systemically assess the cardioprotective effect of GB against DOX-induced cardiotoxicity *in vitro* and *in vivo*.

Currently, the exact mechanisms about DOX-induced cardiotoxicity remains unclear. However, multiple studies have suggested that oxidative stress plays a critical role in the pathogenesis of DOX induced cardiotoxicity [48–50]. DOX induced cellular damage is mediated by an iron-anthracycline complex formation and this complex has the ability to generate free radicals that could cause severe plasma membrane and cytoskeleton structure damage [51]. Due to the lack of effective antioxidant defense mechanisms, anthracycline-induced reactive oxygen species (ROS) could cause severe injury to the heart and because of the central role of ROS in the mechanism of DOX-induced myocardium damage [52], we here examined the anti-oxidative role of GB against DOX-induced toxicity. We observed that GB pretreatment significantly decreased ROS level, which indicates that the protective function of GB against DOX-induced cardiotoxicity is partly due to its anti-oxidative properties.

When cardiomyocytes are subjected to oxidative stress or ischemia reperfusion, the p38MAPK and PI3K/Akt pathways can be altered inside the cells, leading to apoptotic cell death, which subsequently contribute to the deterioration of cardiac contractile function and left ventricular remodeling. Previous studies have suggested that Akt activation could protect heart function by inhibiting cell apoptosis and p38MAPK inhibition could attenuate cellular inflammatory reactions [53]. Moreover, previous investigations indicated that the activation of PI3K/Akt signaling pathway promotes an essential cell survival signaling in cardiomyocytes [54]. Our present findings showed that GB could reduce DOX-induced cardiac cell death by activating the PI3K/Akt signaling pathway. These results are consistent with previous studies [55–57]. In contrast, we did not observe any significant alteration of P38 expression during the GB pretreatment.

In previous studies on ischemia and ischemia-reperfusion induced cardiac arrhythmia, it was demonstrated that GB is able to exert anti-ischemia and cardioprotective effects by inhibiting the increase of the left ventricular end diastolic pressure, improving post ischemia-reperfusion cardiac pump function and protecting the ischemia myocardium from calcium overload reaction due to its negative effect on the intracellular calcium level [58, 59]. Herein, in conformity with the above studies, we equally discovered that GB could decrease the intracellular calcium level and protect from the myocardium damage induced by DOX treatment. Moreover, as previously shown in a mouse model of cardiotoxicity [60], we observed that GB could significantly improve the LVEF and LV mass, thus indicating the substantial effectiveness of GB pretreatment for the body.

## Conclusion

In conclusion, GB exhibits protective effects against DOX-induced cardiotoxicity *in vitro* and *in vivo* by modulating ROS, Akt and calcium pathways. The present findings will contribute in the heart protection during DOX chemotherapy.
